# Rational and Effective Treatment of Hip-Disease

**Published:** 1908-09

**Authors:** 


### BOOKS ON SURGERY.

**Rational and Effective Treatment of Hip-disease.** By P. BRACE BENNIE, M.A., M.D., B.S. (Melb.). Pp. viii., 108. London: Baillière, Tindall and Cox. 1907.

The writer of this book claims to have proved by experience that the only satisfactory methods of treating hip-disease are those of Thomas, and he here describes in great detail those methods, together with certain improvements of them which he has himself introduced. We agree with him that the principles and methods of treatment described by H. O. Thomas are not so well understood by the profession as they should be. Too often the making, fitting and adapting of a Thomas's hip-splint is left to the instrument maker, and the adaptation is imperfect, either from the beginning or later when, with the progress of recovery, a re-adaptation becomes necessary.

It is obvious from the text that Mr. Bennie is a master in the art of applying Thomas's hip-splints and of the principles which underlie their use, and that he has obtained this mastery as a result of prolonged and careful study and practice. It is clear, too, that good results can only be obtained with these splints if they are made and applied with great care. All the details requiring great care are well explained in this little book, and for this reason it cannot fail to be of great use to any surgeons who have hitherto believed that the fitting of the splint could well be left to the instrument maker. We may, however, express the hope that a way will be found of fitting these splints in the best manner possible without resorting to trigonometrical formulæ.

Other forms of splint treatment in addition to that of Thomas are briefly considered, and also operative and constitutional treatment. The observations upon the association of hip-disease with congenital syphilis are specially interesting and suggestive.

The author has done a very real service for the future sufferers from hip-disease, and for the practitioners who treat them, by describing with such fulness and detail his methods of treatment, and showing the excellent results which are obtainable from them.

